# A Peculiar Tooth

**Published:** 1877-07

**Authors:** 


### A PECULIAR TOOTH.

Dr. W. G. Stowel, of Manchester, Michigan, recently sent us a right superior central incisor with a double root.

The bifurcation extends well toward the neck of the tooth. The root at the right side is much the largest, and a little longer than the other, but not the average size of the root of such a tooth. There is a large decay upon the anterior surface of the tooth; the crown appears to have been well formed, and without any marked peculiarity. There was nothing peculiar in any of the other teeth: the full number was present. The person from whom the tooth was extracted was a man about eighteen years old. This is one of the extra processes of nature, for which we have no definite explanation.

